# WQ-3810, a fluoroquinolone with difluoropyridine derivative as the R1 group exerts high potency against quinolone-resistant *Campylobacter jejuni*

**DOI:** 10.1128/spectrum.04322-23

**Published:** 2024-08-20

**Authors:** Kentaro Koide, Hyun Kim, Matthew V. X. Whelan, Lawrence P. Belotindos, Wimonrat Tanomsridachchai, Ruchirada Changkwanyeun, Masaru Usui, Tadhg Ó Cróinín, Jeewan Thapa, Chie Nakajima, Yasuhiko Suzuki

**Affiliations:** 1 Division of Bioresources, Hokkaido University International Institute for Zoonosis Control, Sapporo, Japan; 2 Department of Bacteriology II, National Institute of Infectious Diseases, Musashimurayama, Japan; 3 School of Biomolecular and Biomedical Science, University College Dublin, Belfield, Ireland; 4 Faculty of Public Health, Thammasat University, Pathum Thani, Thailand; 5 School of Veterinary Medicine, Rakuno Gakuen University, Ebetsu, Japan; 6 International Collaboration Unit, Hokkaido University, Sapporo, Japan; 7 Hokkaido University Institute for Vaccine Research and Development, Sapporo, Japan; Innovations Therapeutiques et Resistances, Toulouse, France

**Keywords:** antimicrobial resistance, quinolone, *Campylobacter jejuni*, DNA gyrase

## Abstract

Quinolone-resistant *Campylobacter jejuni* have been increasing worldwide. Quinolones exert their antibacterial activity by inhibiting DNA gyrase, but most of the isolates acquire quinolone resistance via an amino acid substitution in the A subunit of DNA gyrase. WQ-3810 is a quinolone antibiotic that has been reported to have high potency even to DNA gyrase with amino acid substitutions in several bacterial species; however, there was no information on *C. jejuni*. Hence, this study aimed to evaluate the activity of WQ-3810 to inhibit wild-type/mutant DNA gyrases of *C. jejuni* and the bacterial growth for accessing the potency for the treatment of quinolone-resistant *C. jejuni* infection. The inhibitory activity of WQ-3810 was assessed and compared with ciprofloxacin and nalidixic acid by calculating the half maximal inhibitory concentration (IC_50_) against wild-type/mutant DNA gyrases. Next, the minimum inhibitory concentration (MIC) of WQ-3810 and five other quinolones was determined for *C. jejuni* including quinolone-resistant strains with amino acid substitutions in GyrA. Furthermore, the interaction between WQ-3810 and wild-type/mutant DNA gyrase was speculated using docking simulations. The IC_50_ of WQ-3810 against wild-type DNA gyrase was 1.03 µg/mL and not different from that of ciprofloxacin. However, those of WQ-3810 against mutant DNA gyrases were much lower than ciprofloxacin. The MICs of WQ-3810 ranged <0.016–0.031 µg/mL and were the lowest against both quinolone-susceptible and quinolone-resistant strains among the examined quinolones. The results obtained by the docking simulation agreed well with this observation. WQ-3810 seems to be a promising antimicrobial agent for the infections caused by quinolone-resistant *C. jejuni*.

**IMPORTANCE:**

WQ-3810, a relatively new quinolone antibiotic, demonstrates exceptional antibacterial properties against certain pathogens in previous studies. However, its efficacy against quinolone-resistant *Campylobacter jejuni* was not previously reported. The prevalence of quinolone-resistant *C. jejuni* as a cause of foodborne illnesses is increasing, prompting this investigation into the effectiveness of WQ-3810 as a countermeasure. This study revealed high inhibitory activity of WQ-3810 against both wild-type and mutant DNA gyrases of *C. jejuni*. WQ-3810 was equally efficacious as ciprofloxacin against wild-type DNA gyrases but showed superior effectiveness against mutant DNA gyrases. WQ-3810 also demonstrated the lowest minimum inhibitory concentrations, highlighting its enhanced potency against both susceptible and resistant strains of *C. jejuni*. This observation was well supported by the results of the in silico analysis. Consequently, WQ-3810 exhibits a higher level of bactericidal activity compared to existing quinolones in combating both susceptible and resistant *C. jejuni* isolates.

## INTRODUCTION

*Campylobacter* is considered the most frequent bacterial cause of worldwide foodborne gastroenteritis (1). Among the 20 *Campylobacter* species isolated from humans, *Campylobacter jejuni* is the leading causative agent, accounting for 90% of human cases (2, 3). The major symptoms of *C. jejun*i infections include enteritis, abdominal cramps, fever, and diarrhea (4). Most campylobacteriosis in healthy individuals is acute and self-limiting, with the symptoms ceasing within less than 10 days. However, campylobacteriosis can be fatal in some of patients including infants, the elderly, and immunosuppressed persons. Antimicrobial therapy is therefore necessary to achieve the best outcomes during a serious infection. The drug of choice typically includes quinolones because of their high antibacterial activity against *Campylobacter*. One of the most commonly prescribed quinolones is ciprofloxacin, which was selected as an essential medication by the World Health Organization (5). Nevertheless, both developed and developing countries have reported resistance to ciprofloxacin in *C. jejuni* isolates from humans (6, 7). The global escalation of quinolone resistance in *C. jejuni* severely limits therapeutic options and may lead to an increase in severe cases as well as mortality.

The principal mechanism of quinolone resistance in *Campylobacter* consists of a single amino acid substitution in DNA gyrase subunit A (GyrA). DNA gyrase, a tetrameric protein composed of two GyrA and two GyrB, has an enzymatic activity to introduce negative supercoiling into bacterial chromosomes by catalyzing the cleavage and resealing of DNA double strands (8, 9). Quinolones bind to the gyrase-DNA complex and inhibit bacterial replication (10). Amino acid substitutions of GyrA occur in a conserved region of the quinolone binding site, which is called the quinolone resistance-determining region (QRDR) (11). In the QRDR, the mutations responsible for quinolone resistance have been reported mostly at 86th and 90th amino acids, which are considered to be strongly involved in the binding to quinolones (12). In previous reports, amino acid substitution from threonine to isoleucine at position 86 (Thr86Ile) was most frequently identified in quinolone-resistant *C. jejuni* isolates (13). Moreover, Thr86Ile confers a higher level of quinolone resistance than other amino acid substitutions in QRDR (14, 15). Current quinolone treatments for *C. jejuni* infections are much less successful due to the considerable influence of Thr86Ile on quinolone resistance.

In the present study, we aimed to evaluate the potential of WQ-3810 as a therapeutic agent for infections caused by quinolone-resistant *C. jejuni*. WQ-3810 is a relatively new quinolone developed by Wakunaga Pharmaceutical Co., Ltd. In previous studies, high inhibitory activity of WQ-3810 on mutated DNA gyrase was confirmed in a variety of bacterial species other than *C. jejuni* (16–20). WQ-3810 has unique substituents at R1 (6-amino-3,5-difluoropyridine-2-yl) and R7 (3-isopropylaminoazetizine-1-yl). These substituents are thought to significantly enhance its inhibitory effect on mutant DNA gyrases (21, 22). Modifications at the R1 and R7 positions on the quinolone ring may also influence both the pharmacokinetics and toxicity profiles of quinolone antibiotics. It was expected that WQ-3810 would show the high inhibitory activity against *C. jejuni* DNA gyrase with Thr86Ile and exert a favorable antibacterial activity for quinolone-resistant *C. jejuni*. Therefore, we conducted the inhibitory assay of WQ-3810 against DNA gyrases containing Thr86Ile or other mutations as well as the antimicrobial susceptibility tests against *C. jejuni* isolates. Furthermore, we discussed the relationship between the characteristic structure of WQ-3810 and its inhibitory activity against DNA gyrase with Thr86Ile by *in silico* analysis.

## MATERIALS AND METHODS

### Quinolones used in this study

**Fig 1:**
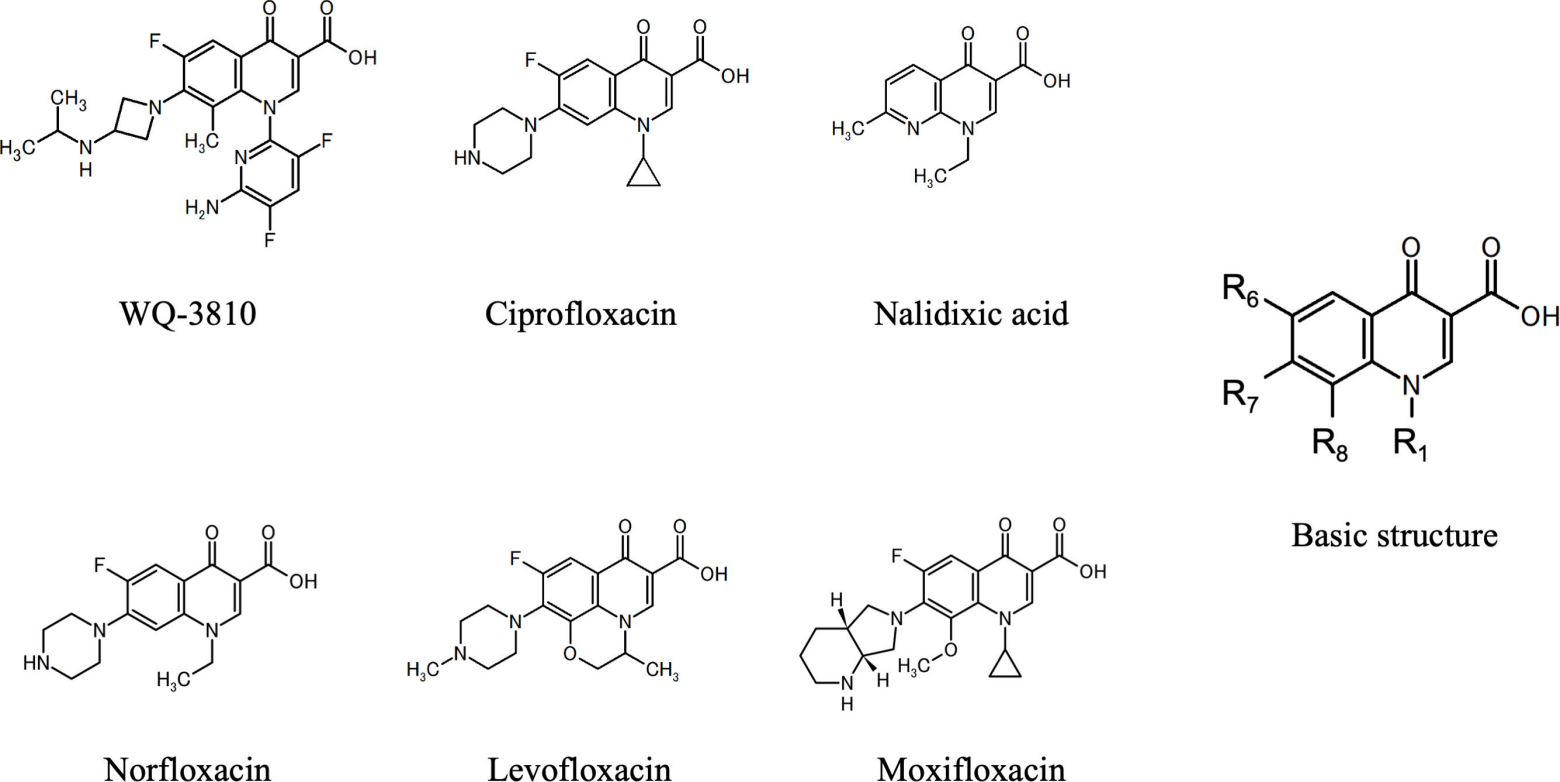
Structures of quinolones used in this study.

WQ-3810 and five quinolones were used in this study. The chemical structures are shown along with a basic structure of quinolone in Fig. 1. WQ-3810 was kindly provided by Wakunaga Pharmaceutical Co., Ltd (Osaka, Japan). Other quinolones were purchased from three companies. Ciprofloxacin and levofloxacin were purchased from LKT Laboratories, Inc. (St. Paul, MN, USA). Nalidixic acid and norfloxacin were purchased from FUJIFILM Wako Pure Chemical Corp. (Osaka, Japan). Moxifloxacin was obtained from Toronto Research Chemicals Inc. (Toronto, ON, Canada). All quinolones were stored according to the manufacturer’s instructions until use.

### Recombinant GyrA and GyrB of *C. jejuni*

Recombinant DNA gyrases were produced to investigate the inhibitory activity of WQ-3810 on DNA gyrase. Seven plasmid vectors coding GyrA or GyrB of *C. jejuni* have been constructed in a previous study (23). Two vectors encoded wild-type (WT) GyrA and GyrB. Others encoded GyrA with the following amino acid substitutions: Thr86Ile, Thr86Lys, Thr86Ala, Asp90Tyr, and Asp90Asn. Using the above seven vectors, recombinant proteins were expressed and purified as described in a previous study with some modification (23, 24). Briefly, each plasmid vector was transformed into *Escherichia coli* BL21(DE3) (Merck KGaA, Darmstadt, Germany), and the transformants were cultured in Luria–Bertani broth (Becton, Dickinson and Company, Franklin Lakes, NJ, USA), containing 100 μg/mL of ampicillin up to log phase. The expression of recombinant protein was then induced by adding 1-mM isopropyl β-D-thiogalactopyranoside(IPTG) and further incubated at 18°C for 16 hours. The harvested cells were lysed by sonication to release the expressed proteins. The recombinant proteins in the supernatant were purified by nickel-nitrilotriacetic acid agarose (Thermo Fisher Scientific Inc., Waltham, MA, USA) column chromatography and dialyzed against DNA gyrase dilution buffer [50-mM Tris-HCl, pH 7.5; 100-mM KCl, 2-mM dithiothreitol (DTT), 1-mM ethylenediaminetetraacetic acid (EDTA)]. The recombinant proteins were mixed with glycerol to a concentration of 40% (wight per volume) to avoid denaturation. The purified protein was confirmed by sodium dodecyl sulfate-polyacrylamide gel electrophoresis (SDS-PAGE).

### Supercoiling inhibitory assay

Supercoiling inhibitory assay using the recombinant DNA gyrases was carried out as previously described (23, 24). Ciprofloxacin and nalidixic acid were employed to compare the inhibitory activity with WQ-3810. Each reaction mixture consisted of 1.5 nM of relaxed pBR322 plasmid DNA (John Innes Enterprises Ltd, Norwich, UK), 36 nM of GyrA and GyrB, 3 mM of ATP in gyrase assay solution [35-mM Tris-HCl, 6-mM MgCl_2_, 1.8-mM spermidine, 24-mM KCl, 5-mM DTT, 0.36-mg/mL BSA, 6.5% glycerol (wt/vol)] with serially diluted quinolone in a total volume of 30 mL. Each mixture was incubated at 37°C for 1 hour, and the enzyme reactions were terminated by adding 8 mL of a stop solution (5% SDS, 25% glycerol, and 0.25 mg/mL bromophenol blue). Then, 10 µL of each mixture was subjected to a 1% agarose gel electrophoresis in 0.5 Tris-borate-EDTA (TBE) buffer. After the gel electrophoresis for 2 hours at 40 mA, the gel was stained for 30 min in 0.5× TBE buffer containing 5 µg/mL of GelRed. Consequently, the band of supercoiled DNA was visualized separately from relaxed DNA under LED light because the supercoiled DNA had a more compact conformation and moved faster in the gel than the relaxed DNA. Each assay was run in triplicate with three quinolones and six DNA gyrases.

To assess the inhibitory activities of quinolones, the half maximal inhibitory concentration (IC_50_) was calculated by plotting concentration–response curves. The brightness of supercoiled DNA band was measured using software ImageJ (http://rsbweb.nih.gov/ij). The measured value of quinolone-free sample was set to 100%, and the brightness of supercoiled DNA bands of other samples was quantified as relative value (%). Concentration–response curve was plotted from all the values, and IC_50_ was calculated by using software R with the add-on package “drc” (25).

### Bacterial strains and culture conditions

A total of 12 strains were used to evaluate the antimicrobial activity of WQ-3810 against both quinolone-susceptible and quinolone-resistant *C. jejuni*. One of them was a type strain *C. jejuni* ATCC 33560, which is known as a quality control strain for antimicrobial susceptibility testing (26). Five quinolone-resistant strains were obtained by culturing the type strain on the growth medium containing quinolones. The other six strains were *C. jejuni* isolated from raw chicken meat purchased at supermarkets in Sapporo, Japan. *C. jejuni* were routinely cultured on mueller-hinton (MH) agar in a tri-gas incubator at 37°C with microaerophilic conditions (N_2_: 85%, CO_2_: 10%, and O_2_: 5%).

To select strains with quinolone resistance, *C. jejuni* ATCC 33560 was cultured on MH agar supplemented with increasing concentration of ciprofloxacin or nalidixic acid. Ciprofloxacin was supplemented in the MH agar at 0.1 µg/mL and increased to a maximum concentration of 100 µg/mL through 0.3, 1, 3, 10, and 30 µg/mL. Single colonies were picked up at 1-, 10-, and 100-µg/mL ciprofloxacin and termed C1, C2 and C3, respectively. Nalidixic acid in MH agar were 10, 50, 100, and 300 µg/mL for the stepwise selection, and two mutant strains were collected from MH agars containing 10- and 300-µg/mL nalidixic acid and named N1 and N2, respectively.

### DNA sequencing and sequence analysis

Nucleotide substitution in the QRDR of *gyrA* gene was confirmed by polymerase chain reaction (PCR) and sequencing. The PCRs of the forward and reverse primers were 5′-AGAGATGGTTTAAAGCCTGTTC-3′ (nucleotide position 120–142) and 5′-ACGCCCTGTGCGATAAGCTTC-3′ (nucleotide position 700–720), respectively. The amplification was conducted under the following conditions: predenaturation at 95°C for 1 min, 30 cycles of denaturation at 95°C for 10 sec, annealing at 47°C for 10 sec and extension at 72°C for 30 sec, and a final extension at 72°C for 5 min. The nucleotide sequences of the PCR products were determined using BigDye Terminator Cycle Sequencing Kit version 3.1 and ABI Prism 3130xl Gene Analyzer (Thermo Fisher Scientific Inc.). Specific genomic mutations in the QRDR associated with quinolone resistance were identified by comparing obtained sequences with the reference genome of *C. jejuni* (RefSeq: NC_002163.1) using MEGA X (https://www.megasoftware.net/).

### Antimicrobial susceptibility testing

The susceptibility of the *C. jejuni* strains to six quinolones was examined by broth microdilution method. The minimum inhibitory concentration (MIC) was determined to assess growth inhibition exhibited by each quinolone according to the criteria of the Clinical and Laboratory Standards Institute (CLSI) (27). Each strain was cultured on MH agar plate for 48 hours, and multiple colonies were then suspended in MH broth. The turbidity of the bacterial suspension was adjusted with 0.5 McFarland standards. An adjusted bacterial inoculum was added to each well of a sterile 96-well plate containing the test concentrations of quinolones. After the incubation for 48 hours at 37°C, the lowest concentration at which no bacterial growth was observed with the naked eye was designated as MIC. This examination was run in triplicate for each strain by quinolone to confirm reproducibility.

### *In silico* analysis of the molecular interaction between DNA gyrase and quinolone

Molecular docking simulation was performed to identify the correct pose of WQ-3810 in the binding site of DNA gyrase. Interaction between WQ-3810 and DNA gyrase of *C. jejuni* was analyzed by using Molecular Operating Environment (MOE) software (Chemical Computing Group ULC, Montreal, Quebec, Canada. https://www. chemcomp.com/index.htm). The 3D-structure model of DNA gyrase is necessary to implement the simulation, but that of *C. jejuni* has not been classified yet. Therefore, a homology model for *C. jejuni* DNA gyrase has been built based on ID 6RKS registered in Protein Data Bank (https://www.rcsb.org/structure/6RKS). This is a crystal structure model of *E. coli* DNA Gyrase with DNA binding. The amino acid sequence of QRDR of *E. coli* has high similarity with that of *C. jejuni* (28). Amino acid substitution of Thr86Ile was introduced into the model by Coot release 0.8.9.1 (https://www2.mrc-lmb.cam.ac.uk/personal/pemsley/coot/). Ligand location was calculated based on QRDR by Coot as well. The pose of WQ-3810 in the binding site of DNA gyrase was estimated by calculating the binding free energy of DNA gyrase and WQ-3810 using MOE software with the default parameters of Amber 10: EHT force field. Same analysis was performed for ciprofloxacin as comparator.

Results of docking simulation were visualized using PyMOL 2.4 (http://www.pymol.org/), and the distances between amino acid residues and WQ-3810 were calculated using Coot. Previous study showed intermolecular force such as a hydrogen bond played an important role in the molecular interaction between quinolone and DNA gyrase (29). This is a distance-dependent interaction between molecules. The distance of hydrogen bonds is generally considered to be from 2.5 to 3.2 angstroms (Å). Therefore, distances greater than 4 Å were excluded from the analysis, as too long distance between the quinolone and the amino acid residue is unlikely to be involved in the interaction.

## RESULTS

### IC_50_ of WQ-3810, ciprofloxacin, and nalidixic acid

**Fig 2:**
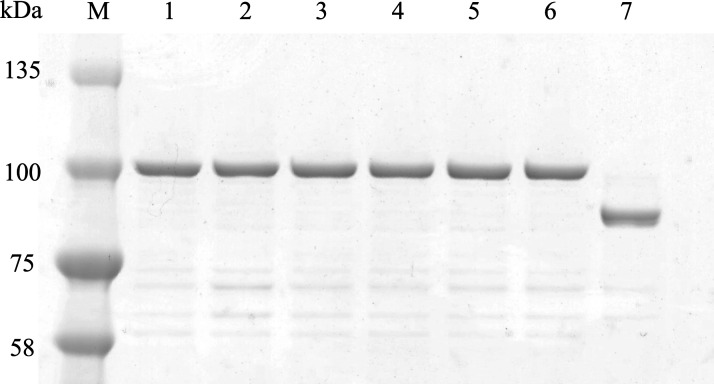
SDS-PAGE analysis of purified GyrA and GyrB. Three hundred nanogram of each purified protein was loaded onto a 5%–20% gradient polyacrylamide gel. High-purity purification of GyrA and GyrB at approximately 97 and 87 kDa, respectively, was confirmed. Lanes: M, protein marker; 1, WT-GyrA; 2, Thr86Ile-GyrA; 3, Thr86Lys-GyrA; 4, Thr86Ala-GyrA; 5, Asp90Asn-GyrA; 6, Asp90Tyr-GyrA; and 7, WT-GyrB.

**Fig 3:**
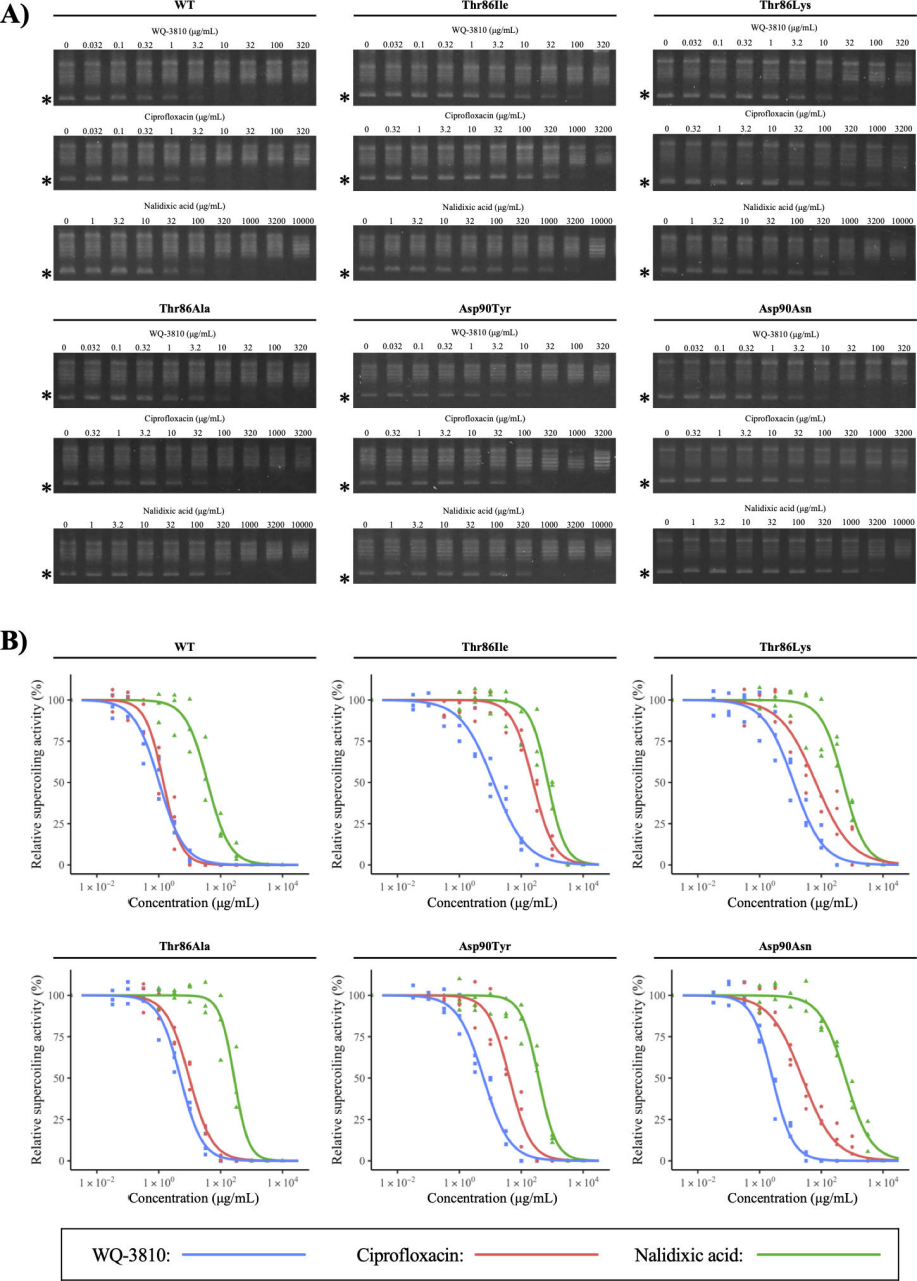
Results of supercoiling inhibitory assay. (**A**) Gel electrophoretic image measuring the inhibitory activity by quinolone. The position of the supercoiled DNA band is indicated by an asterisk (*) on the left of the electrophoresis images. These figures are randomly selected from a triplicate assay. (**B**) Concentration–response relationships of WQ-3810, ciprofloxacin, and nalidixic acid are shown. One hundred percent of supercoiling activity was defined as the supercoiling activity in quinolone-free samples. The relative values of the supercoiling activity inhibited by WQ-3810, ciprofloxacin, and nalidixic acid at each concentration were plotted as blue-, red-, and green-filled circles, respectively. Since each assay was performed in triplicate by quinolone, there are three respective measured values at each concentration. The concentration–response curve for each quinolone was drawn in its corresponding color.

The inhibitory effect of quinolones was assessed by using recombinant DNA gyrases. The high purity of recombinant GyrA and GyrB of *C. jejuni* was identified by SDS-PAGE at 97 and 89 kDa, respectively (Fig. 2). Using the recombinant DNA gyrases and quinolones, the concentration-dependent inhibitory activities of quinolones on DNA gyrase were confirmed by gel electrophoresis (Fig. 3A). Afterward, the brightness of DNA bands attenuated by three quinolones was measured, and the curves plotted from all measured values are shown in Fig. 3B. The horizontal axis shows the concentration of quinolone, while the vertical axis represents the relative supercoiling activity of DNA gyrase compared to the activity of DNA gyrase in the quinolone-free sample. Nalidixic acid required higher concentrations to reduce the amount of supercoiled DNA for all WT and mutant DNA gyrases, positioning its curves at the most right. On the other hand, the curves for WQ-3810 appeared to the left of other curves for the mutant DNA gyrases, indicating its efficacy at lower concentrations. The only exception was that the curves of WQ-3810 and ciprofloxacin for WT DNA gyrase overlapped.

The IC_50_ was calculated by plotting concentration–response curves to assess the inhibitory activities of quinolones. The IC_50_ of each quinolone was summarized in Table 1. As confirmed in Fig. 3B, the highest IC_50_ for all WT and mutated DNA gyrases was observed for nalidixic acid. Comparing ciprofloxacin and WQ-3810, there was no significant difference of IC_50_ in WT DNA gyrase, while IC_50_ of WQ-3810 for mutated DNA gyrases was lower than those of ciprofloxacin. For ciprofloxacin, the highest IC_50_ was 241 µg/mL, and it was confirmed in DNA gyrase with Thr86Ile. The IC_50_ of WQ-3810 for DNA gyrase with Thr86Ile was also highest among those of other mutated DNA gyrases, but the value was 12.8 µg/mL, which was about 1/20 of the concentration needed with ciprofloxacin. Additionally, the second highest IC_50_ was confirmed in DNA gyrase with Thr86Lys. The IC_50_ of WQ-3810 for DNA gyrase with Thr86Lys was about 1/5 that of ciprofloxacin. There was no significant difference in IC_50_ of WQ-3810 between DNA gyrases with Thr86Ile and Thr86Lys although IC_50_ of ciprofloxacin for DNA gyrase with Thr86Ile was 3.6-fold higher than that for DNA gyrase with Thr86Lys.

**TABLE 1:** IC_50_ of WQ-3810, ciprofloxacin, and nalidixic acid for WT and mutant DNA gyrases^*a*^

GyrA	IC_50_ (95% CI) (μg/mL)		
	WQ-3810	Ciprofloxacin	Nalidixic acid
WT	1.03 (0.794–1.26)	1.41 (1.13–1.68)	37.8 (29.8–45.8)
Thr86Ile	12.8 (8.91–16.6)	241 (183–298)	724 (562–885)
Thr86Lys	12.6 (8.59–16.7)	66.3 (42.7–89.9)	519 (371–666)
Thr86Ala	5.04 (4.06–6.01)	9.70 (7.78–11.6)	273 (229–317)
Asp90Tyr	6.03 (4.61–7.45)	41.6 (32.8–50.5)	365 (294–435)
Asp90Asn	2.61 (2.13–3.09)	24.2 (18.7–29.8)	611 (488–734)

^*a*^ Note: All results have been rounded to no more than three significant figures.

### MICs of WQ-3810 and five quinolones

The MIC of WQ-3810 was lower than that of other quinolones against both quinolone-susceptible and quinolone-resistant strains. Amino acid substitutions in QRDR of each strain and MICs of six quinolones were summarized in Table 2. The C257T (Thr86Ile) and G268A (Asp90Asn) were detected in lab strains produced by the selection using ciprofloxacin and nalidixic acid, respectively, and only C257T (Thr86Ile) was identified in strains isolated from chicken. All the strains having amino acid substitution in GyrA were categorized as quinolone resistance because the MICs were equal or above 4 µg/mL which is the break point for resistance to ciprofloxacin according to the CLSI criteria (30). Among currently available quinolones, the MIC of moxifloxacin was the lowest against all strains examined in this study. However, the MIC of WQ-3810 was about 1/65 to 1/2 of that of moxifloxacin. The MICs of quinolones other than WQ-3810 against high-level resistant strain C3 were more than 16 µg/mL, whereas the MIC of WQ-3810 against C3 was 2.0 µg/mL.

**TABLE 2:** MICs of WQ-3810 and other quinolones

Strain	Amino acid substitution inQRDR in GyrA	MIC (μg/mL)					
		WQ-3810	Ciprofloxacin	Nalidixic acid	Norfloxacin	Levofloxacin	Moxifloxacin
Type strain							
ATCC 33560	−	0.016	0.25	4–8^*a*^	0.5	0.25	0.125
Lab strains							
N1	Asp90Asn	0.031	4	128	16	4	2
N2	Asp90Asn	0.063	8	256	16	4	2
C1	Thr86Ile	1	8	64	16	16	4
C2	Thr86Ile	2	32	128	32	16	8
C3	Thr86Ile	2	> 32	256	32	32	16
Isolated strains							
H2-1	−	< 0.016	0.125	4	0.5	0.25	0.031
MN11-2	−	0.031	0.25	8	1	0.5	0.25
M1-63	−	0.031	0.5	16	2	0.5	0.25
M2-10	Thr86Ile	1	16	128	32	8	2
M12-16	Thr86Ile	1	16	128	32	8	2
H1-9	Thr86Ile	1	16	256	32	8	2

^*a*^ To check for reproducibility, each test was performed in triplicate, but only two results were obtained for the MIC of nalidixic acid in ATCC33560: 4 and 8 mg/L.

### Simulated interaction between WQ-3810 and DNA gyrase

**Fig 4:**
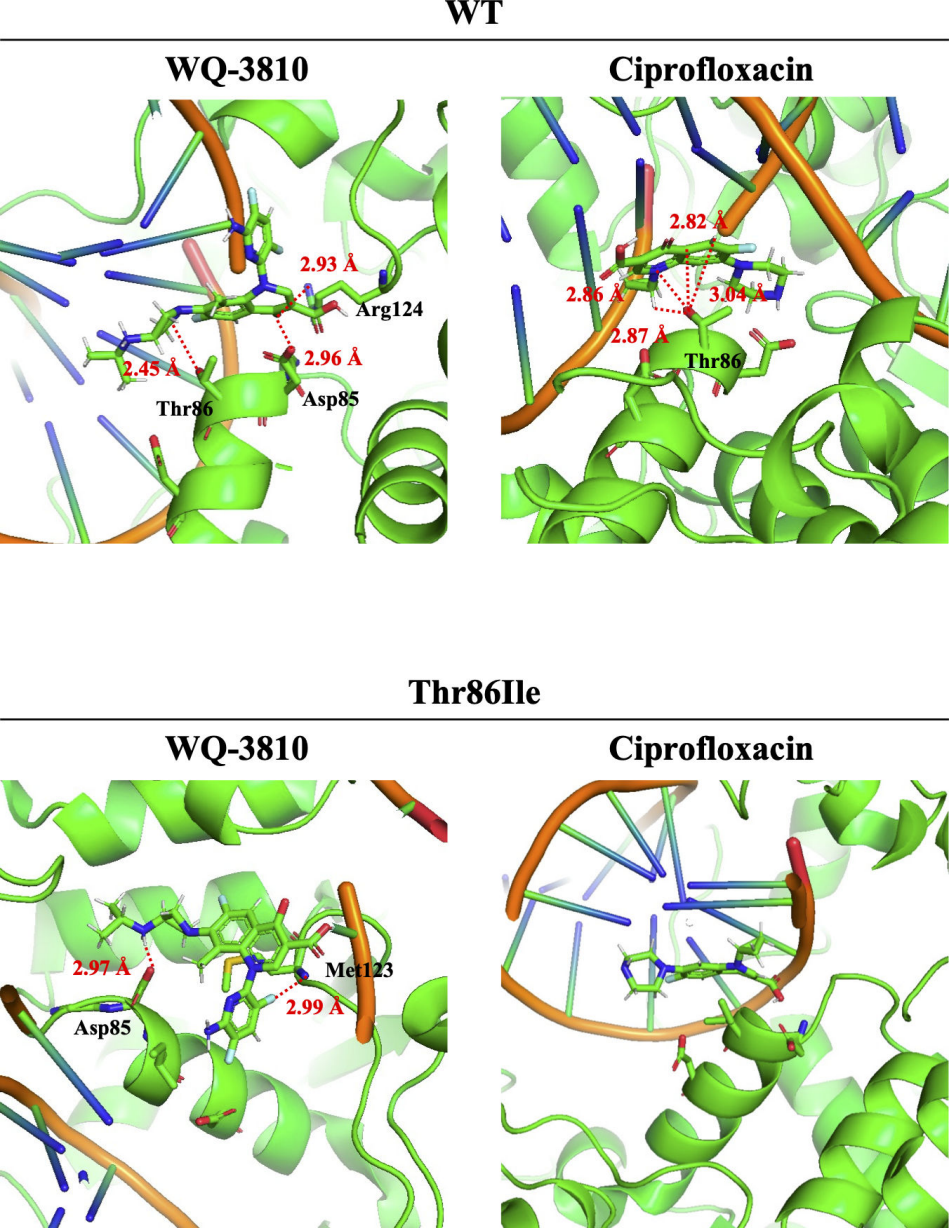
Molecular interaction between DNA gyrase and quinolones. Green cartoon ribbon indicates the protein secondary structure and orange lines are DNA motif. The amino acids involved in binding and their positions in the amino acid sequence of *C. jejuni* are shown in black letters. the distances between the amino acid residues and the quinolone are indicated by red dotted lines as well as red letters.

The binding formation of WQ-3810 in the pocket of DNA gyrase was different from that of ciprofloxacin. The results of docking simulation were visualized in Fig. 4. The 3D structure of the binding pocket was computationally built from the amino acid sequences of GyrA and GyrB of *C. jejuni* based on ID 6RKS of the DNA gyrase of *E. coli* registered in Protein Data Bank. In the figure, the amino acid positions represent the positions in the amino acid sequence of *C. jejuni*. Ciprofloxacin interacted only with the threonine at position 86 of WT GyrA (Thr-86), whereas interactions with multiple amino acid residues were observed for WQ-3810. WQ-3810 was adjacent to aspartic acid at position 85 (Asp-85) as well as Thr-86. In addition, WQ-3810 also interacted with arginine which was at position 124 of the other subunit of the two GyrAs. In the simulation model of DNA gyrase with Thr86Ile, ciprofloxacin was located far from any amino acid residue in GyrA. On the other hand, WQ-3810 maintained a close distance to two amino acid residues of GyrA. The R1 and R7 substituents of WQ-3810 were in close proximity to Asp-85 and methionine at position 123, respectively.

## DISCUSSION

There is a great need for new antibiotics to treat infections caused by quinolone-resistant *C. jejuni*. To investigate the potential of WQ-3810 as a therapeutic agent, the principal focus of this study was on the inhibitory effect of WQ-3810 on *C. jejuni* DNA gyrase with Thr86Ile. Ciprofloxacin and other quinolones bind to DNA gyrase by scaffolding the 86th amino acid on GyrA, whereas WQ-3810 is anticipated to interact with amino acids at a different position compared to currently used quinolones, via R1 and R7 substituents on the quinolone ring (16, 18). It suggested that Thr86Ile does not have a significant influence on the binding affinity of WQ-3810 to DNA gyrase and the high antibacterial activity against quinolone-resistant *C. jejuni* was expected. However, previous studies showed less bactericidal effect of WQ-3810 compared to other quinolones although WQ-3810 exerted high inhibitory effect on DNA gyrases (17, 18). This is thought to be due to poor permeability to the bacterial cell wall of some bacterial species, and its effect on *C. jejuni* is unclear. Therefore, supercoiling inhibitory assay and antimicrobial susceptibility test, as well as docking simulations, were all carried out to evaluate the antibacterial activity of WQ-3810 against quinolone-resistant *C. jejuni* in this study. The outcomes of these three experiments indicate whether WQ-3810 is effective against quinolone-resistant *C. jejuni* and may have implications for future quinolone development.

The supercoiling inhibitory assay showed a promising inhibitory effect of WQ-3810 on the mutated DNA gyrases of *C. jejuni*. Nalidixic acid and ciprofloxacin were used in comparison, for the inhibitory assay. As nalidixic acid is a quinolone which used to screen quinolone-resistant isolates in certain pathogens (31, 32), there was a considerable difference in IC_50_ of nalidixic acid between WT and mutant DNA gyrases. It indicated that amino acid substitutions on GyrA were responsible for the increased IC_50_ as previously reported (23). Among the tested amino acid substitutions, the highest ciprofloxacin resistance was found in DNA gyrase with Thr86Ile. WQ-3810 inhibited the activity of DNA gyrase with Thr86Ile at lower concentration than ciprofloxacin. Furthermore, there was no difference in the IC_50_ of WQ-3810 between DNA gyrase with Thr86Ile and Thr86Lys in contrast to ciprofloxacin. Isoleucine is a hydrophobic residue, whereas threonine and lysine are both hydrophilic amino acids. Thr86Ile is an amino acid substitution that leads to the polarity shift, and it may weaken the hydrogen bonding between DNA gyrase and ciprofloxacin more than Thr86Lys (23). However, the inhibitory activity of WQ-3810 was hardly affected by changes in polarity. This suggests that the DNA gyrase and WQ-3810 interact with amino acids at positions other than Thr-86. Also, WQ-3810 inhibited the supercoiling activity of DNA gyrase with an amino acid substitution at position 90 at lower concentrations than ciprofloxacin. It was determined that regardless of the polarity and position of the amino acid substitution on GyrA, WQ-3810 had greater inhibitory effect against mutant DNA gyrases than ciprofloxacin.

The antimicrobial susceptibility testing was performed with *C. jejuni* strains with three different levels of quinolone resistance: quinolone-susceptible, intermediate-level quinolone resistance, and high-level quinolone resistance. Of 12 strains used in this study, eight strains were categorized as quinolone-resistant strains. The amino acid substitutions detected in the quinolone-resistant strains were either Thr86Ile or Asp90Asn. As Thr86Ile conferred higher quinolone resistance on DNA gyrase than Asp90Asn in the supercoiling inhibitory assay, the MICs of all the tested quinolones except nalidixic acid were higher in quinolone-resistant strains with Thr86Ile. All isolated strains possess Thr86Ile on GyrA, and the level of quinolone resistance was intermediate in accordance with previous studies (33, 34). Furthermore, even for strains with the same amino acid substitution, MICs differed among them. This may be caused by the development of additional antimicrobial resistance mechanisms, such as efflux pumps (35). As a result of the accumulation of its resistance mechanisms, strain C3 having DNA gyrase with Thr86Ile showed the highest-level quinolone resistance. The antimicrobial activity of WQ-3810 against quinolone-resistant *C. jejuni* was investigated using moderately quinolone-resistant strains isolated from chicken meat, a potential source of infection, and highly quinolone-resistant strains that may emerge in the future.

High antibacterial activity of WQ-3810 was observed against both quinolone-susceptible and quinolone-resistant *C. jejuni* strains. A type strain and three strains isolated from chicken had WT DNA gyrase and were categorized as quinolone-susceptible. MICs of WQ-3810 against the quinolone-susceptible strains were lower than ciprofloxacin, and despite the result of supercoiling inhibitory assay, there was no significant difference in IC_50_ against WT DNA gyrase between the two quinolones. Although WQ-3810 was reported to have low antibacterial activity due to its poor permeability (17, 18), this study demonstrated that WQ-3810 has an advantageous antibacterial activity against quinolone-susceptible *C. jejuni* compared to ciprofloxacin. This advantage, together with its high-level inhibition for DNA gyrase, enables WQ-3810 to effectively inhibit the growth of quinolone-resistant *C. jejuni* strains as well. Among the currently available quinolones tested in this study, MICs of moxifloxacin were the lowest for all strains. It is noteworthy that the MIC of WQ-3810 was much lower than moxifloxacin against both the quinolone-resistant strains isolated from chicken meats and the high-level quinolone-resistant strain of C3. It is summarized that WQ-3810 is more effective against both quinolone-susceptible and quinolone-resistant *C. jejuni* than available quinolones, signifying that WQ-3810 has high therapeutic utility.

The results obtained by *in vitro* assays was well supported by *in silico* analysis. The docking simulation showed that Thr86Ile caused the different binding manner of quinolone to DNA gyrase. The binding of ciprofloxacin to WT DNA gyrase was mediated by Thr-86. By substitution from threonine to isoleucine, ciprofloxacin lost its anchor point for binding to DNA gyrase. As noted in the results of the supercoiling inhibitory assay, the polarity of the amino acid residues was considered to be an important factor for the direct interaction between quinolone and amino acid residues in GyrA. The side chains of amino acid, in particular, contribute to intermolecular forces with drugs through hydrogen bonding and electrostatic interactions (36). The chemical formula of threonine is C_4_H_9_NO_3_ (molecular weight: 119.12), and it is an amino acid with a polar functional group on its side chain. Isoleucine (C_6_H_13_NO_2_, molecular weight: 131.17), on the other hand, has two more carbons than threonine and is a nonpolar amino acid. These features may modify the distance between quinolone and amino acid residue and also cause the decreased binding affinity of ciprofloxacin to DNA gyrase. Besides, *in silico* analysis showed that WQ-3810 has multiple interactions with amino acids in the binding pocket via R1 and R7 of the quinolone structure, which may minimize the effect of the aforementioned amino acid substitutions on GyrA. It is known that the R7 substituent has a significant effect on the antibacterial activity of quinolone (10, 37, 38), but in WQ-3810, not only R7 but also R1 play an important role in the inhibitory activity on DNA gyrase. The present study revealed that the high inhibitory effect of WQ-3810 on DNA gyrase with Thr86Ile would be attributed to the interaction of R1 and R7 with amino acid residues that have not been reported to be substituted in the binding pocket.

In conclusion, WQ-3810 showed high inhibitory activity on DNA gyrases with amino acid substitution. WQ-3810 forms multiple bonds with amino acid residues in the binding pocket via substituents of R1 and R7, thereby making the binding affinity less susceptible to amino acid substitutions conferring quinolone resistance. This may lead the high antibacterial activity of WQ-3810 against quinolone-resistant strains as well. The present study demonstrated that WQ-3810 exerted higher bactericidal activity than currently available quinolones against both quinolone-susceptible and quinolone-resistant *C. jejuni*. Therefore, WQ-3810 is a promising candidate for treatment of foodborne diseases caused by *C. jejuni*.
